# Factors Affecting the Ability of the Stroke Survivor to Drive Their Own Recovery outside of Therapy during Inpatient Stroke Rehabilitation

**DOI:** 10.1155/2014/626538

**Published:** 2014-03-27

**Authors:** Xue Wen Eng, Sandra G. Brauer, Suzanne S. Kuys, Matthew Lord, Kathryn S. Hayward

**Affiliations:** ^1^Division of Physiotherapy, School of Health and Rehabilitation Sciences, University of Queensland, Brisbane 4072, Queensland, Australia; ^2^Griffith Health Institute, Griffith University, Gold Coast 4222, Queensland, Australia; ^3^Allied Health Research Collaborative, Metro North Hospital and Health Service, Brisbane 4132, Queensland, Australia

## Abstract

*Aim*. To explore factors affecting the ability of the stroke survivor to drive their own recovery outside of therapy during inpatient rehabilitation. *Method*. One-on-one, in-depth interviews with stroke survivors (*n* = 7) and their main carer (*n* = 6), along with two focus groups with clinical staff (*n* = 20). Data was thematically analysed according to group. *Results*. Stroke survivors perceived “dealing with loss,” whilst concurrently “building motivation and hope” for recovery affected their ability to drive their own recovery outside of therapy. In addition, they reported a “lack of opportunities” outside of therapy, with subsequent time described as “dead and wasted.” Main carers perceived stroke survivors felt “out of control … at everyone's mercy” and lacked knowledge of “what to do and why” outside of therapy. Clinical staff perceived the stroke survivor's ability to drive their own recovery was limited by the lack of “another place to go” and the “passive rehab culture and environment.” *Discussion*. To enable the stroke survivor to drive their own recovery outside of therapy, there is a need to increase opportunities for practice and promote active engagement. Suggested strategies include building the stroke survivor's motivation and knowledge, creating an enriched environment, and developing daily routines to provide structure outside of therapy time.

## 1. Introduction

Stroke is the world's third most common cause of long-lasting disability [1]. Accordingly, the need for effective and efficient inpatient rehabilitation for stroke survivors cannot be underestimated. Intensity of practice has been consistently highlighted as a critical component of therapy provided during inpatient rehabilitation [2]. A higher intensity, in terms of minutes or repetitions of practice, has been found to promote greater functional gains during inpatient rehabilitation than less intensive therapy [3–5]. This has led researchers and therapists to direct greater attention to the creation of opportunities for intensive practice to drive recovery during therapy time, such as alternate models of care for example, group circuit classes or seven-day therapy service [6], and the use of technology including virtual reality and gaming [7] and robotic therapy [8]. However, less attention has been directed to the creation of opportunities to drive recovery outside of therapy.

Studies to date indicate that stroke survivors may not use time outside of therapy optimally during inpatient rehabilitation. A recent systematic review [9] of observational studies from around the world (including Europe, United Kingdom, and Australia) reported that stroke survivors consistently spend the majority of time outside of therapy inactive (median 48.1% of the day), alone (median 53.7% of the day), and in their bedroom (median 56.5% of day). Thus, to increase the intensity of practice undertaken during inpatient rehabilitation, there is substantial scope to improve how time is used outside of therapy.

One approach to increase activity levels outside of therapy is to create an enriched environment. A recent study found that an enriched inpatient rehabilitation environment can promote greater activity levels and reduce alone time as compared to a nonenriched environment [10]. This study also highlighted observed barriers to increase activity levels outside of therapy, which included the inability of the stroke survivor to mobilise without assistance, hospital processes and routines that may discourage physical activity to compensate for low staff numbers, and the lack of access to occupational and physiotherapy gyms during nontherapy hours and on weekends. However, there remains little evidence from the perspective of the stroke survivors regarding factors that may influence their ability to use time outside of therapy to drive their own recovery. Further, the changes to physical and psychological capabilities after stroke can limit the stroke survivor's confidence [11] and capacity [12] to take responsibility of their own recovery. As a result, their main carer and clinical staff (including nurses, physiotherapy, occupational therapy, and speech therapy) often play an integral role in their recovery during inpatient rehabilitation. This demonstrates the need to explore the unique perspectives of each of these three groups. Thus, the aim of this study was to explore factors affecting the ability of the stroke survivor to drive their own recovery outside therapy within inpatient stroke rehabilitation, from the perspective of the stroke survivor, their main carer, and the clinical staff.

## 2. Methods

A qualitative research design was chosen to gain new insights and to allow for a holistic consideration of possible factors that may affect the ability of the stroke survivor to drive their own recovery outside of therapy during inpatient rehabilitation. Ethical approval was obtained from the relevant hospital and university medical research ethics committees and the study was completed in accordance with the Declaration of Helsinki.

### 2.1. Setting

The study was completed within a four-ward geriatric and rehabilitation unit within a tertiary metropolitan hospital in Queensland, Australia. The majority of stroke survivors are allocated to one of two 26-bed general rehabilitation wards. Each ward has four to six nursing staff per shift, who, respectively, manage four to six beds. Each ward has approximately 2 to 2.5 full-time equivalent physiotherapists, 1.5 to 2 full-time equivalent occupational therapists, and 0.5 to 1 full-time equivalent speech language therapist, who manage approximately 10 patients each. In general, stroke survivors receive therapy for one to three hours per day across the three disciplines, within geographically separate and discipline specific areas. No after hours or weekend therapy is offered.

### 2.2. Participants

Consecutively admitted patients with a diagnosis of stroke were invited to participate if they were medically stable (confirmed by the medical registrar), receiving physiotherapy, occupational therapy, or speech language therapy during inpatient rehabilitation, and were able to follow single stage commands. Each stroke survivor nominated a main carer, who was defined as the person they spent the most time with while in hospital. All clinical staff (nursing staff, physiotherapists, occupational therapists, and speech pathology) of the two wards were invited to participate. No exclusion criteria were imposed for stroke survivors, carers, or staff. All participants provided written informed consent. Participant recruitment ceased upon saturation of the data, which was deemed to be the point where no additional information was added to enhance or distinguish emerging concepts within each group.

### 2.3. Procedure

In-depth interviews and focus groups were conducted and voice-recorded. In depth, one-on-one interviews were completed with stroke survivors and main carers to encourage comfortable sharing of personal information and individual experiences and to facilitate individualised probing of emerging themes. In contrast, focus groups were completed with clinical staff to stimulate an information-rich group discussion between the varying disciplines and levels of clinical experience. Interviews were arranged and conducted by one facilitator (XWE) on one occasion. Two focus group discussions were arranged to enable maximum participation across disciplines and were conducted by one facilitator (SK), supported by a scribe who took comprehensive field notes on group dynamics, nonverbal language, and emerging themes of the discussion. Each focus group included up to 10 clinical staff. All facilitators were independent of the hospital and thus were not involved in stroke survivor care or clinical staff supervision. Interviews and focus groups were allocated a 1-hour block and were conducted within a private research laboratory located off the wards to provide a neutral environment.

Four open-ended stimulus questions (Table 1) were used for all interviews and focus group discussions. In these discussions, the term function was used to encapsulate the ICF domains of impairment, activity, and participation [13]. At the commencement of each discussion, the facilitator explained the study purpose and distributed interview questions. Participants were invited to immediately write down their thoughts to each question to capture their unbiased and spontaneous thoughts and perspectives and to encourage participants to subsequently contribute in their own words. The facilitator then asked each of the four key questions. There was no strict adherence to the style and type of questioning beyond these four key questions, with probes used to explore or challenge emerging themes, personal experiences, and ideas. All discussions were drawn to a close with the facilitator summarizing the main points raised, in which participants were then provided with the opportunity to add or dispute what had been said or contribute any final thoughts.

### 2.4. Analysis

Analysis occurred continuously throughout the study. During data collection, the facilitator (and scribe for the focus groups) immediately reflected on the dialogue after each discussion to document what might be considered the “group data” and to ensure that what had been learnt could be used to extend questioning and challenge subsequent discussions. All audio recordings of the discussions were transcribed verbatim (XWE) and were cross-checked by another researcher (ML) against the audio record to verify accuracy.

An approach consistent with conventional thematic content analysis was used [14]. On completion of data collection, the transcripts of each participant group were explored separately through a process of reading and rereading. Two researchers, one of whom was not involved in data collection, independently reviewed all transcripts within their allocated participant group (stroke survivor: XWE, KH; main carers: XWE, SK; and clinical staff: XWE, SB). On the first reading, transcripts were read in their entirety to acquire a whole sense of the data. On the second reading, line-by-line analysis was used to identify themes, patterns, or concepts and any factors affecting the ability of the stroke survivors to drive their own recovery outside of therapy were listed. This led to the tentative collation of predominant themes within each group. The third reading of the data was used to check the fit of the themes with the transcripts, pursuing patterns or concepts that were both consistent and inconsistent with the data. The original themes were then modified as required to more appropriately represent the data. Subsequently, all reviewers met to discuss the group themes and any overlap or mismatch between groups. At this point, the conditions under which each theme arose and its relationship with other themes (within and between groups) were documented. Finally, a member check was completed with participants using group-specific data. All findings are reported in group-specific language where possible to ensure the relevance to each group was maintained.

Literature was reviewed to establish the need for the study. In-depth review of the literature as per the identified themes which occurred after commitment to the themes was established. This was done to ensure that the themes emerged from the data and to limit the impact of any preconceived categories or ideas that may be present in the literature.

## 3. Results

A total of 33 participants were recruited: seven stroke survivors, six carers, and 20 clinical staff, comprising eight (40%) nurses, eight (40%) physiotherapists, three (15%) occupational therapists, and one (5%) speech language therapist. Demographics of each participant group are summarized in Table 2. One stroke survivor approached declined to participate; however, their nominated carer did consent; and two included stroke survivors did not nominate a main carer. One-on-one interviews lasted between 15 minutes and 1 hour depending on the responsiveness of the interviewee. Focus group discussions lasted between 45 minutes and 1 hour. Outcomes of the member check resulted in minor clarification of terms and focus of themes, which ensured they were directed at the stroke survivor's ability to drive their own recovery.

**Figure 1:**
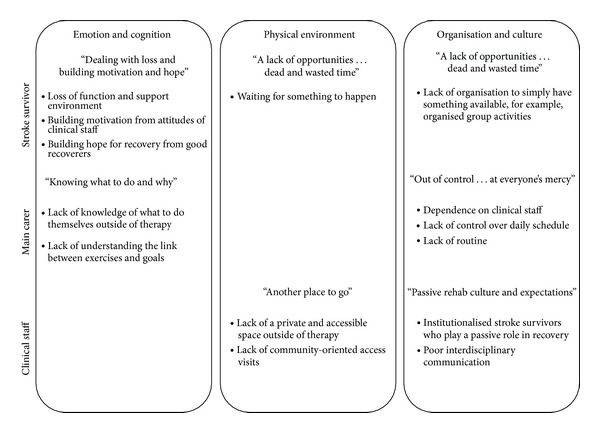
Factors affecting the ability of the stroke survivor to drive their own recovery outside of therapy within inpatient stroke rehabilitation.

Across all participant groups, the fundamental factor expressed to positively affect the stroke survivor's ability to drive recovery of function outside of therapy after stroke was a seamless “continuation of the same tasks” undertaken inside of therapy to outside of therapy. This was described as physical tasks such as “doing therapy” and “just practicing … doing things for themselves.” This highlights the fact that time outside of therapy was perceived as an opportunity to undertake task practice within real world environments. The themes that emerged in each group could largely be divided into three overarching categories: (1) emotion and cognition, (2) physical environment, and (3) organisation and culture (Figure 1). Individual themes identified by each group will be discussed in more detail.

### 3.1. Stroke Survivor's Perspective

#### 3.1.1. Dealing with Loss and Building Motivation and Hope

Stroke survivors attributed a lot of their idle and inactive “downtime” outside of therapy to result from dealing with the grief of having a stroke and resulting loss of function. As a result of their stroke, they felt “pulled out of their normal support environment,” with many comments on the lack of physical and emotional contact for support through the early stages after stroke. Dealing with loss was described to influence their capacity to drive their own recovery.

In comparison “building motivation” to drive recovery was described as heavily influenced by the attitudes of the clinical staff, which is an external source. A loss of motivation was described to occur in response to “doctors being negative about their poor rate of recovery when they extended their estimated length of stay,” which prevented them from returning home as early as anticipated. On the contrary, positive staff attitudes that acknowledged their progress were reported to strongly encourage and build motivation so as to persevere with recovery outside of therapy.

Another significant source of encouragement to drive recovery was “building hope” for recovery. A contributor to this process was described to stem from witnessing firsthand recovery achieved by stroke survivors further down the track. Interacting with other stroke survivors was a strong source of motivation and hope for many interviewed. It provided reinforcement of the potential outcomes of putting in hard work to drive their own recovery, which they often acknowledged would require independent practice outside of therapy.

#### 3.1.2. A Lack of Opportunities … Dead and Wasted Time

Stroke survivors defined a large proportion of their time outside of therapy as “dead and wasted.” This was especially so on weekends, where much time was spent simply “waiting for something to happen.” Despite feeling that they needed “to be doing something more” and acknowledging that “it was easy to be complacent,” they strongly perceived it was the responsibility of the clinical staff to provide such opportunities. Stroke survivors rarely attempted to create their own opportunities for practice.

In line with this, stroke survivors perceived there to be a lack of organisation by the unit. They expressed the need to simply “have something available” and “organised” at least once daily (weekday and weekends), with a strong focus towards “more organised group activities.” Suggestions ranged from group craft or cooking classes, through to a “general fitness program” or scheduled walking practice. Access to clinical staff at these times was indicated to be highly favoured for guidance, instruction, and safety. Furthermore, they perceived that having “exercise equipment accessible in the bedroom” could also enable and support physical practice. In spite of this, stroke survivors expressed the need to balance physical practice with rest and relaxation.

### 3.2. Main Carer's Perspective

#### 3.2.1. Out of Control … at Everyone's Mercy

Carers perceived stroke survivors felt “out of control over their own recovery.” This was considered to be largely due to their loss of physical independence and resulting dependence on clinical staff, especially nurses for activities of daily living. However, they also highlighted the frequent unpunctuality of the clinical staff as another significant contributor to feeling out of control, as it often translated to a lack of adherence to the planned daily schedule. Carers perceived stroke survivors consequently felt stressed and “exhausted just from waiting around,” which results in “unsettled rest” before therapy even started. In addition, this waiting to “be shunted around all day” was thought to establish a sense of “not knowing what they were doing” and a mindset that they had no control over their own daily schedule. This mindset was seen to extend over to the time outside of therapy, which was perceived to have a negative impact on their ability to drive their own recovery.

*“They say … How can I help with my recovery when I'm at everyone's mercy to tell me if or when?”*

The concept of routine and structure was repeatedly raised by carers as key to facilitate a stroke survivor to drive their own recovery outside of therapy. This was perceived to encourage “a continuation of this really structured program” inside of therapy to outside of therapy. Suggestions to achieve this included introducing “more informative” routine into the stroke survivors day through scheduling of exercise time, rest time, meal time, and so forth or laying out a clear structure of what to do and when, for example, “written checklist of tasks they could tick off,” such as “ward exercises for the different positions patients spent a lot of time in” to maximise time spent in and out of bed. In addition, they perceived that stroke survivors would benefit from engagement in discussions with clinical staff to develop specific patient-oriented goals that are linked to daily tasks and training to enable them to undertake these tasks outside of therapy.

#### 3.2.2. Knowing What to Do and Why

Carers raised a lack of knowledge as a key hindrance to stroke survivors driving their own recovery outside of therapy. Not knowing “what they could be doing themselves” outside of therapy and the “ambiguous” limits and guidelines unaddressed by therapy-related clinical staff of whether they should “push some more” were viewed as particularly limiting.

*“They say … I'll do anything to get out of here, but I just don't know what to do.”*

In essence, carers perceived the role of the clinical staff as fundamental to equip the stroke survivor with the knowledge and understanding of what they could do independently outside of therapy, for example, exercises. Carers also emphasized that therapy-related clinical staff in particular needed to provide clear, step-by-step, explicit education of how the prescribed independent exercises were “relevant to daily tasks.” Developing the stroke survivors understanding of how the exercises would help to “get them home” was regarded to be crucial, together with an emphasis on urgency to maximise the “window of opportunity” for recovery.

### 3.3. Clinical Staff Perspective

#### 3.3.1. Another Place to Go

Clinical staff regarded the unit to have the potential to be a “positive environment” for facilitating stroke survivors to drive their own recovery outside of therapy. The daily incidental mobility and activity of daily living practice from having to access the dining room at least thrice daily was regarded as extra beneficial physical “rehabilitation” outside of therapy. On the flipside, a significant environmental barrier raised across all disciplines was the lack of opportunity to access “another place to go” outside of therapy, which was dedicated, private, and accessible for independent practice of exercises or therapy homework.

On top of environmental factors within the rehabilitation unit, clinical staff also emphasised the importance of more community-oriented activities for progressive exposure and interaction of the “isolated stroke survivors within the community.” In order to simultaneously encourage the beneficial “physical practice of walking and buying coffee,” for instance, staff suggested either having “more actual community access visits” to a nearby public cafe or having “something from the outside world come into the rehabilitation unit, such as a mobile coffee cart.” Such examples would provide greater opportunity for real world task practice.

#### 3.3.2. Passive Rehab Culture and Expectations

Clinical staff perceived that the medical model of health care results in institutionalized stroke survivors who expect and/or are willing to play a passive role in rehabilitation.

*“But do we have a culture where the expectation is “I'm in hospital. I go and sit by my bed. And I just go off to things when people call for me” … Is that something that we need to address?”*

The clinical staff acknowledged their part to play in reinforcing this passive culture. Nurses were seen to provide excessive physical assistance, while therapists expressed a lack of quality time for discussions about goals, ward exercises, or involving and enlisting the help of carers. Time constraints were blamed for this across clinical staff disciplines, resulting in the perceived need for “more staffing to meet national guidelines,” extra therapy assistants or volunteers, or even greater involvement of carers.

Clinical staff unanimously regarded the key solution to this deeply ingrained problem to be better interdisciplinary team communication to establish specific patient-oriented team goals. Nurses expressed the value of therapists communicating the stroke survivor's specific current goal through the use of “rehab diaries or the patient's bed boards,” so they could then, for instance, “give the (stroke survivor) time to try to put on their shirt independently.” Another suggestion to improve communication was the use of the Unit's patient journey board, which outlines their daily schedule to timetable in homework or exercise time outside of therapy. This way nursing staff could schedule time to be available to assist the stroke survivor if required.

## 4. Discussion

This study sought to identify factors affecting the ability of the stroke survivor to drive their own recovery outside of therapy within inpatient rehabilitation from the perspective of the stroke survivor, their main carer, and clinical staff. From all three perspectives explored, it was consistently highlighted that time outside of therapy may be underexploited. From the stroke survivor's perspective, time outside of therapy was described as “dead and wasted.” Their main carers reflected on the stroke survivors lack of “knowing what to do and why” as a possible contributor, whilst clinical staff commented on the lack of “another place to go” and the contribution of the “passive rehab culture and expectations.” Also highlighted in this study were the emotional consequences of stroke. Stroke survivors are “dealing with loss” after stroke, whilst concurrently “building motivation and hope,” which carers perceived to be compounded by stroke survivors being “out of control … at everyone's mercy.” These findings have critical implications for future studies that attempt to build opportunities for stroke survivors to drive their own recovery outside of therapy and self-manage the consequences of stroke.

Stroke survivors described that a part of their time outside of therapy is directed towards dealing with their loss. Stroke can have an enormous impact on a person's life. In a previous metasynthesis of studies [15], stroke survivors were found to describe stroke as a sudden and overwhelming catastrophe that changes their life irrevocably (with a significant impact on activities, roles, and social relationships). Furthermore, stroke survivors have previously highlighted the ongoing struggle after stroke to adjust and regain a sense of self [16]. Stroke survivors of this study also echoed the impact of dealing with loss after stroke. During inpatient rehabilitation, this suggests that clinical staff may need to balance the focus on intensive practice with providing time to deal with the emotional consequences of stroke and grieve the sudden loss imposed on the stroke survivors' lifestyle.

“Building motivation” to drive recovery outside of therapy also emerged across groups. Interestingly, in this study, stroke survivors highlighted their reliance on external sources for motivation, for example, realistic feedback on progress and encouragement from clinical staff. In contrast, clinical staff expressed that high levels of internal motivation were critical to a stroke survivor's ability to drive their own recovery. This demonstrates a disparity between what stroke survivors and clinical staff perceive as the key source of motivation after stroke (external versus internal). Such a mismatch may be linked to a lack of understanding and knowledge, as main carers expressed that the transference of knowledge (of what and why) from an external source was required to enable stroke survivors to drive their own recovery. A previous study of stroke survivors has also highlighted a possible link between motivation and understanding and knowledge of rehabilitation [17]. This points to the need for individually tailored education of stroke survivors and their main carer by clinical staff. Further, it suggests that to improve motivation, education will need to extend beyond the provision of didactic information to implementation and practical training. This is consistent with a self-management approach [11, 18], which is receiving growing attention in the chronic phase of stroke recovery. Yet akin to previous studies [16], stroke survivors of this study appeared to find the self-manager role challenging, which indicates that greater focus on development of these skills during the subacute phase of recovery may be indicated.

A key factor expressed across all three groups was a lack of* opportunity* to drive their own recovery outside of therapy. Stroke survivors highlighted that they experienced a lot of “dead and wasted time” outside of therapy, while clinical staff perceived there was a lack of “another place to go” for therapeutic purposes outside of therapy. According to previous observational studies during inpatient rehabilitation, stroke survivors spend a large proportion of time inactive, alone, and in their bedroom [9]. Therefore, while the current study supports previous findings that stroke survivors are often inactive and alone, a point of difference from previous observational studies is that the outcomes of this study demonstrated the feeling associated with inactive time, that is, “dead and wasted,” and highlighted suggestions of how to possibly address this issue from a variety of perspectives, for example, creating opportunities for practice. This suggests there is a need to investigate how to change the physical environment to provide greater opportunities for practice outside of therapy.

One method to enhance the physical environment that is receiving growing attention is an enriched rehabilitation environment. A recent study by Janssen and colleagues [10] found that an enriched environment (defined by additional heightened motor, sensory, cognitive, and social stimulation) can be used to provide greater opportunities for independent practice outside of therapy after stroke and reduce alone and inactive time. What constitutes an enriched environment clinically is still ambiguous though, as methods used in past animal model studies have varied widely and appear difficult to emulate in a clinical setting [19, 20]. This current study adds several suggestions as to what the three groups perceived may beneficially enrich the environment, including exercise equipment accessible in the bedroom to enable and support physical practice, organizational supports such as a “to do checklist” or list of things to do when alone and inactive, the provision of structured therapy homework that can be completed in their room or within a dedicated and accessible private space, and the opportunity for real world engagement such as going to have a coffee. By involving stroke survivors in the planning to enrich their environment, it may also serve to increase the onus on the stroke survivor to manage their time outside of therapy, thus reducing the passive rehabilitation culture.

In order to achieve maximum benefit from a physical environmental change, it would appear there is a need to improve the environment with regard to organisation, communication, and culture within inpatient rehabilitation. After stroke, there is very little that the stroke survivor, who was often premorbidly independently functioning, has control over. Carers described it as “out of control … at everyone's mercy.” Carers interviewed indicated that stroke survivors would benefit from engagement in discussions with clinical staff to develop specific patient-oriented goals that are linked to daily tasks and training to enable them to undertake these tasks outside of therapy. Consistent with this and as evident in stroke guidelines [21], it would appear that one of the first things that needs to occur on admission to an inpatient rehabilitation ward is the development of shared goals, which was also highlighted by a recent metasynthesis of stroke survivors views of the impact of stroke [16]. Extending from this is the need to develop a daily routine or schedule through active communication between the stroke survivor, their carer, and the clinical staff to promote achievement of the goal. Critical elements of such a routine may include therapy time, independent exercise time, rest time, and social time. Development of a daily routine will aid in the promotion of good habits, which have been previously highlighted to be important for achievement of good recovery after stroke [22, 23]. Through building routine, it may also serve to build the stroke survivors capacity to take ownership of how their day runs. Such skills have the potential to serve stroke survivors and their carers well in their long-term quest for recovery beyond inpatient rehabilitation. Commitment to the developed routines will require improved communication between clinical staff and the stroke survivor and their main carer. This demonstrates the integral role of a team approach to the organisation of inpatient rehabilitation.

The strengths and limitations of this study need to be considered to place these findings in perspective. As a whole, the stroke survivors recruited were fairly representative of the inpatient stroke rehabilitation population, in terms of mean age and time since stroke. In an attempt to achieve maximum variation in our sample, the views of two younger stroke survivors were captured, two participants did not have a main carer, one participant had two main carers, and one participant had English as a second language. However, a limitation in variation is the large proportion (6 of 7) of stroke survivors recruited who had a right-sided stroke lesion. Future studies should look to explore the perspectives of stroke survivors with both left- and right-sided lesions but concurrently ensure those with communication difficulties can be involved in an in-depth interview. The one participant with a left-sided lesion did find it difficult to communicate her views due to her expressive aphasia. With regard to carers, there was variability in age, relationship to the patient, and amount of time spent on the ward, and the clinical staff included members of four different disciplines and a wide range of ages and levels of clinical and stroke rehabilitation experience. This provides for a diverse sample population and subsequent insights. While all researchers involved had a physiotherapy background, there was a spread in clinical and research experience, ranging from undergraduate students to greater than 20-years experience. A key limitation of the current study design, however, is that it involved a single site. This would suggest that the environmental and organisational factors raised might be specific to this site and their rehabilitation environment layout. But given the consistent findings internationally with regard to how time is spent outside of therapy (inactive, alone, and in the bedroom), it is plausible that similar factors would arise elsewhere. In addition, the current findings arose despite weekly case conferences to facilitate interdisciplinary communication and from a clinical staff group who were well experienced in providing stroke rehabilitation (average 9-years stroke experience). Therefore, the possible solutions to improve the environment and culture to enable stroke survivors to drive their own recovery outside of therapy seem relevant for other sites. Finally, the views expressed are group specific. Future studies should look to investigate the interplay across groups through mixed group discussions with stroke survivors, carers, and clinical staff, with a variety of clinical backgrounds leading the discussions. This may result in shared recommendations to enrich the rehabilitation culture and environment and support the stroke survivors to drive their own recovery outside of therapy during inpatient rehabilitation.

*Clinical Implications.* Several implications for clinical practice have emerged from this qualitative study. Firstly, there is a need to balance intensive rehabilitation with emotional support and management of stroke survivors and carers during the inpatient rehabilitation phase after stroke. Secondly, there is a need for greater emphasis to be directed at building shared goals, achieved through active communication between clinical staff and stroke survivors (and carers). This may also facilitate the development of a daily routine for stroke survivors during inpatient rehabilitation and the identification of opportunities for practice that promote a continuation of tasks completed inside of therapy to outside of therapy. Finally, there is a need to provide individually tailored education to stroke survivors as well as their carers, which goes beyond didactic information to include strategies for implementation and practical training.

## 5. Conclusion

Enabling stroke survivors to drive their own recovery outside of therapy within inpatient rehabilitation is a crucial first step in preparing them to drive their recovery in the long term. This may be achieved by building the stroke survivor's motivation and knowledge, creating an enriched rehabilitation environment, and developing daily routines to increase structure to time spent outside of therapy.

## Figures and Tables

**Table 1:** Key discussion questions for in-depth interviews and focus groups.

(1) What do you think promotes recovery of function after stroke during inpatient rehabilitation?	
(2) What do you think promotes recovery of function outside of therapy?	
(3) What factors do you think influence your/the stroke survivor's ability to drive their own recovery outside of therapy?	
(4) How do you think your/the stroke survivor's ability to drive their own recovery outside of therapy can be maximised?	

**Table 2:** Participant demographic details by group.

	Stroke survivor (*n* = 7)	Main carer (*n* = 6)	Clinical staff (*n* = 20)
Age (mean, SD)	59, 18	58, 15	39, 11
Gender, male (*n*)	4	2	1
Days since stroke (mean, SD)	28, 37	—	—
Stroke affected upper limb, left (*n*)	6	—	—
Dominant upper limb affected, yes (*n*)	1	—	—
Employment status, working at onset (*n*)	3	5	—
Main carer residing with stroke survivor before stroke, yes (*n*)	—	3	—
Hours main carer spent per week with stroke survivor during hospitalisation (mean, SD)	—	25, 30.4	—
Minutes main carer spent in one-way travel to hospital (mean, SD)	—	29, 18.0	—
Years of clinical experience (mean, SD)	—	—	13, 9.5
Years of stroke clinical experience (mean, SD)	—	—	9, 6.0
